# Establishment and culture of mouse oviductal organoids and isolation and characterization of their secreted extracellular vesicles

**DOI:** 10.1371/journal.pone.0337587

**Published:** 2025-12-04

**Authors:** Riley E. Thompson-Brandhagen, Mindy A. Meyers, Richard B. McCosh, Fiona K. Hollinshead

**Affiliations:** 1 Department of Clinical Sciences, Colorado State University, Fort Collins, Colorado, United States of America; 2 Animal Reproduction and Biotechnology Laboratory, Colorado State University, Fort Collins, Colorado, United States of America; 3 Department of Biomedical Sciences, Colorado State University, Fort Collins, Colorado, United States of America; Rocky Vista University and Midwestern University, UNITED STATES OF AMERICA

## Abstract

Oviductal (fallopian tube) cell cultures are widely used to improve understanding of oviductal pathophysiology and to improve sperm and embryo quality via co-culture. One type of oviductal cell culture is organoids, a three-dimensional cell culture that can mimic the oviduct *in vitro* long-term (months). Because organoids maintain their structure and function similar to *in vivo*, their secretions tend to be more similar to *in vivo* cellular products. One of these organoid secretions includes extracellular vesicles (EVs), which are bioactive nanoparticles secreted by cells for intercellular communication. Here we report a protocol for isolation of mouse oviducts, establishment and long-term culture of mouse oviductal organoids, and isolation and characterization of the EVs secreted by mouse oviductal organoids. This lab protocol can be used for research of oviductal physiology and pathology and improvement of assisted reproductive technologies.

## Introduction

Cell culture is widely used in biological research to reduce animal use and to evaluate a tissue directly. Most commonly, monolayer and explant cell cultures are implemented. Monolayers proliferate long-term but lose their normal function often after one passage, and explants maintain their function but are viable in culture for short periods (typically within 2–5 days) [1]. Organoids are a type three-dimensional cell culture that can proliferate long-term (months to years) while maintaining their original phenotype and molecular characteristics [2]. Organoids have gained more interest in the past several years with many groups attempting to incorporate these three-dimensional, physiologically representative *in vitro* cell cultures into their research programs. Because every species and tissue type has different requirements, culture conditions must be evaluated for every new species and/or tissue type, including the types of supplements in the culture medium that support proliferation and how the cells are isolated from the original tissue. For example, intestinal, liver, endometrial, and trophoblast organoids proliferate in culture medium with distinct supplements for each tissue type [3–6], and the culture condition requirements are different for endometrial organoids from mice versus women with mouse endometrial organoids needing WNT3A supplemented in the culture medium while human endometrial organoids do not require WNT3A but do require RSPO1 supplementation [7].

Mouse oviductal (fallopian tube) organoids have been established from each anatomical section of the oviduct, including the infundibulum, ampulla, and isthmus, and the organoids derived from the distal oviduct (the infundibulum/fimbria) are more enriched with stem cells that allow better organoid proliferation [8]. The organoid culture in the lab protocol provided is distinct from the article above because the present article does not separate the oviduct into segments and instead uses cells from the whole oviduct due to the limitations in growth when growing organoids from the separate sections, does not use fluorescence-activated cell sorting (FACS) to isolate the cells prior to organoid generation, and the organoid culture medium supplements are different than those previously described for mouse oviductal organoids [8]. Some experiments involving oviductal organoids have included using a transwell insert to co-incubate human oviductal organoids with sperm to improve sperm motility [9], evaluating the cellular origin of high grade serous ovarian carcinoma using mouse oviductal organoids [10], and investigating the oviductal molecular response to heat stress in bovines [11]. Another research area that is gaining interest involves secretions produced by oviductal organoids, including extracellular vesicles (EVs) [1].

EVs are nanoparticles with a lipid bilayer containing a bioactive cargo of proteins, lipids, nucleic acids, glycans, metabolites, and other small molecules that are secreted by cells for intercellular communication [12,13]. EVs from the reproductive tract have been used for various applications, including the improvement of ARTs. For example, EVs have been demonstrated to improve *in vitro* maturation (IVM) of gametes and *in vitro* culture (IVC) of embryos [14–16]. EVs from amniotic cells co-cultured with bovine embryos during IVC improve expanded blastocyst hatching and pregnancy rates [14], and oviductal fluid contains EVs that interact with gametes/embryos to initiate successful pregnancy [15,16]. One study described how supplementation with bovine oviductal EVs, which were isolated from oviductal fluid, improved embryo development and quality [17], and a recent study demonstrated that EVs isolated from oviductal fluid of women following hysterectomy resulted in improved murine blastocyst and hatching rate [18]. However, an alternative source of *in vitro*-derived oviductal EVs would increase their implementation in ARTs, as flushing oviducts post-mortem using slaughterhouse tissues can only be used in the species that undergo routine slaughter as a source of food. Furthermore, slaughterhouse tissue may not be a repeatable source of EVs as the history of the animal is limited which may impact the cargo of the EVs. Antemortem flushing of oviducts may be possible in most species, however, surgical or laparoscopic oviductal flushing is an invasive process. Oviductal EVs produced *in vitro* would be a non-invasive, repeatable, and sustainable source of EVs. Because 3D cell cultures are more similar to *in vivo* tissue than many other cell culture methods, such as monolayers, their EVs tend to be more similar to those produced *in vivo* [19]. Therefore, EVs produced by oviductal organoids may provide a sustainable, repeatable, and physiologically-relevant source of EVs to improve ART outcomes in various species, including women, mice, and domestic animal species.

The provided lab protocol can be used for collecting mouse oviducts surgically for organoid culture, establishing oviductal organoid culture, and maintaining oviductal organoids long-term (months). This protocol also provides information for how to collect and process oviductal organoids for downstream applications including RT-qPCR, histology, and transmission electron microscopy (TEM). Furthermore, the isolation of EVs secreted by oviductal organoids into the culture medium and their characterization using nanoparticle tracking analysis (NTA), western blot, and TEM, are detailed in this protocol.

## Materials and methods

The lab protocol described here in this peer-reviewed article is published on protocols.io, https://dx.doi.org/10.17504/protocols.io.8epv5kzz4v1b/v1 and is included for printing as S1 File with this article and incorporates detailed ‘tips’ to maximize successful implementation of the lab protocol.

Procedures were approved by the Colorado State University Institutional Animal Care and Use Committee (#3960, PI McCosh). Prior to experimentation, mice were housed with a 12 h light/12 h dark light cycle and had free access to feed and water in accordance with the Guide for the Care and Use of Laboratory Animals in a Colorado State University vivarium (RRID: SCR_022157). Data from adult female C57BL/6 mice (9–16 weeks old) are included in representative data. Mice were anesthetized using isoflurane anesthesia (1−5% isoflurane in medical grade oxygen), and their eyes were lubricated to protect the cornea from damage. Buprenorphine (0.012–0.016 mg/kg) was administered before the start of surgery for analgesia. Ovariectomy was performed via mid-lateral laparotomy under the isoflurane anesthesia using aseptic technique with periodic monitoring for depth of anesthesia. No animals were sacrificed for these studies, and all animals recovered uneventfully post-surgery.

## Expected results and discussion

**Fig 1:**
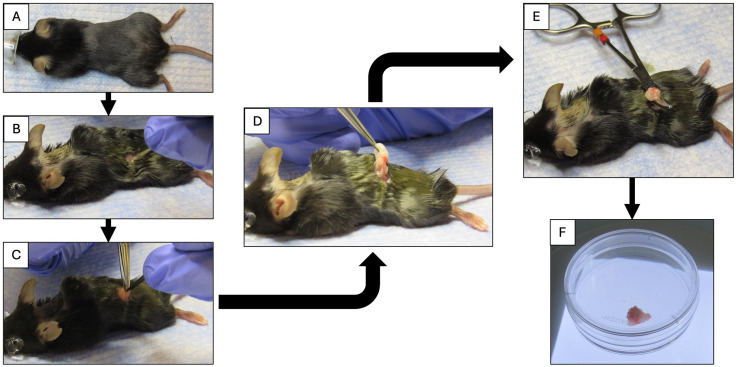
Surgical collection of oviducts. **A)** Anesthetized mouse with eye lubricant applied and fur clipped for ovariectomy; **B)** Dorsolateral skin incision in lumbar area (located halfway between bottom of rib cage and hip); **C)** Incision through body wall; **D)** Externalization of fat pad containing ovary and oviduct; **E)** Hemostatic forceps clamped between uterus and oviduct; **F)** Isolated ovary, oviduct, and adipose tissue in culture dish.

**Fig 2:**
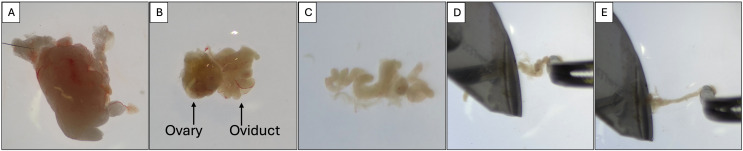
Isolation of oviducts. **A)** Ovary, oviducts, and adipose tissue after surgical isolation; **B)** Ovary with oviduct coiled to its right after dissection from adipose tissue; **C)** Oviduct extended following removal from ovary; **D** and **E)** Scraping and squeezing oviduct with scalpel blade while stabilizing oviduct with thumb forceps.

**Fig 3:**
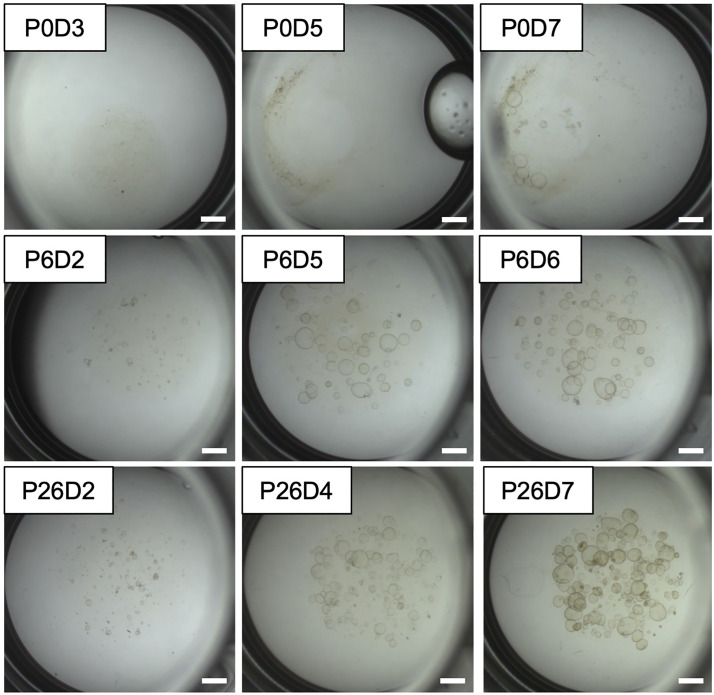
Brightfield micrographs of mouse oviductal organoids throughout culture. P = passage; D = day. Scale bar = 1 mm.

**Fig 4:**
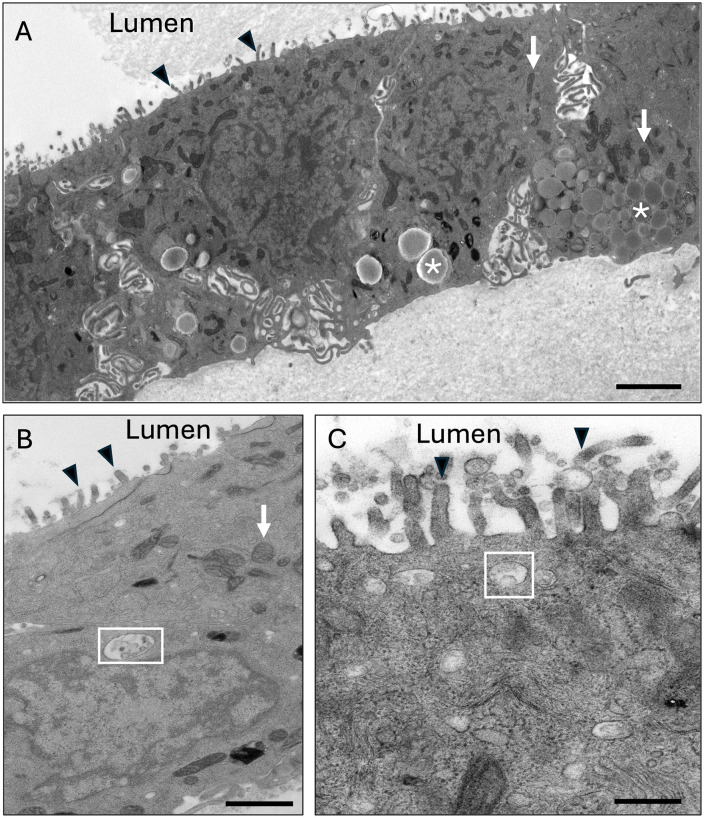
Transmission electron micrograph (TEM) of mouse oviductal organoid at passage 17, day 7 (P17D7). Black arrowheads indicate microvilli; white boxes indicate multivesicular bodies containing extracellular vesicles; white arrows indicate mitochondria; * indicate lipid droplets,. Scale bars = 2 μm **(A)**, 1 μm **(B)**, 400 nm **(C)**.

**Fig 5:**
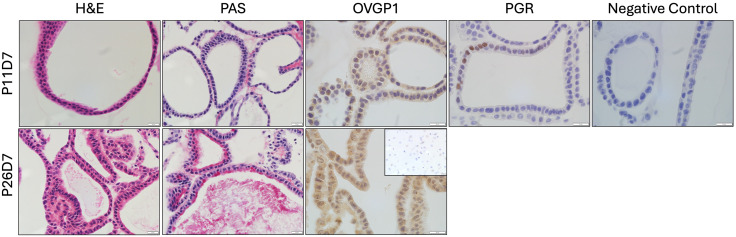
Histological and immunohistochemical (IHC) micrographs of mouse oviductal organoids at passage 11, day 7 (P11D7) and P27D7. Micrographs include hematoxylin and eosin (H&E), periodic acid-Schiff (PAS) staining, and IHC for OVGP1 (Proteintech #22324-1-AP; 1:500 dilution) and progesterone receptor (PGR; ThermoFisher Scientific #MA5-14505; 1:400 dilution). Inset shows tissue negative control (mouse brain) for OVGP1 (1:500 dilution). Scale bars = 20 μm.

**Fig 6:**
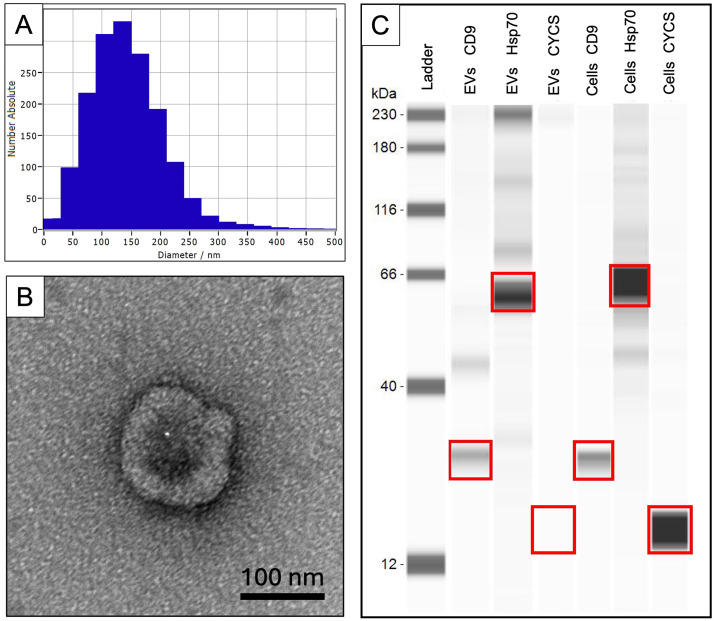
Characterization of extracellular vesicles (EVs) secreted by mouse oviductal organoids. **A)** Nanoparticle tracking analysis (NTA) output illustrating the size distribution of the EVs. **B)** Transmission electron micrograph (TEM) of a single EV showing diameter and morphology; scale bar = 100 nm. **C)** Jess Simple western blot demonstrating presence of EV markers CD9 and Hsp70 and absence of the cell specific protein cytochrome C (CYCS).

Fig 1 shows the step-by-step procedure to surgically collect mouse oviducts, and Fig 2 demonstrates isolation of each oviduct. Figs 3-6 demonstrate the expected outcomes when following this lab protocol. Because mouse oviductal organoids have poor proliferation if cells are isolated from the proximal oviductal segments [8], the protocol provided here does not section the oviduct into gross anatomical segments prior to cell culture. Fig 3 contains brightfield micrographs of mouse oviductal organoids in culture from the first week of initial establishment through passage 26, day 7 (P26D7) which totals 187 days in culture, and the images depict organoid growth and proliferation during P0, P6, and P26 using the described protocol. Mouse oviductal organoid morphology shows round, multi-cellular structures that are light in color with a central area that is brighter than the edges, indicating presence of lumen, i.e., a ‘cystic’ morphology. The organoids maintain this morphology throughout the over 6-month culture period when adhering to the provided lab protocol.

The downstream applications of organoids include the same assays that can be performed on other types of cell culture, including RT-qPCR, histology [20], immunohistochemistry [20,21], and TEM [22]. Fig 4 contains transmission electron micrographs of a mouse oviductal organoid which portrays the expected ultrastructure. Microvilli are present along the apical surface of the cells, indicating polarity toward the central organoid lumen (Fig 4A-C). Other structures include mitochondria and lipid droplets, particularly along the basal aspect of the cells (Fig 4A and B), and presence of multivesicular bodies containing small EVs (exosomes; Fig 4B and C). Fig 5 contains histological and immunohistochemical micrographs of mouse oviductal organoids demonstrating the organoid structure as a layer of cells surrounding a central lumen. Furthermore, positive periodic acid-Schiff staining at passage 11, day 7 (P11D7) and P26D7 indicates active mucin secretion (Fig 5). Additionally, organoids from both passages demonstrated positive staining for OVGP1, an oviduct-specific protein, and positive staining for progesterone receptor was demonstrated in P11D7 organoids but not P26D7. Positive staining for hormone receptors may be more likely if organoids are treated with exogenous steroid hormones similar to the estrous cycle, as previously described [20].

Following isolation via differential ultracentrifugation (see Protocol Step 6 in S1 File), EVs secreted by mouse oviductal organoids should undergo basic characterization using the standards established in the *Minimal Information for Studies of Extracellular Vesicles* [23]. These include establishing the i) size and concentration, such as via nanoparticle tracking analysis (NTA), ii) morphology, such as using TEM [24], and iii) presence of EV proteins and absence of cellular proteins, such as through western blot (Fig 6). A representative graph showing the EV size distribution is depicted in Fig 6A, and Table 1 shows data derived from the NTA from five replicates of isolated EVs from mouse oviductal organoid conditioned medium. The data presented in Table 1 includes the original volume of conditioned medium, the volume after EV isolation, the concentration after EV isolation, and the total EVs in the sample which was calculated by multiplying the volume of isolated EVs (mL) and concentration (particles/mL). The conditioned medium was pooled across organoid passages, as indicated in Table 1, as representative samples from various periods throughout the long-term culture. Table 1 also indicates that the median EV size ranged from 108 nm to 139 nm (Table 1), and this size was confirmed with TEM (Fig 6B). The characteristic ‘cup shaped’ morphology also is demonstrated in Fig 6B. A report of EVs flushed from the mouse oviduct demonstrated presence of the EV tetraspanin protein CD9 and the cytosolic protein Hsp70 [25], and the EVs collected from the spent medium of mouse oviductal organoids also demonstrated presence of these proteins (Fig 6C) and absence of the cellular protein CYCS, indicating that no cellular proteins are contaminating the EV preparation.

**Table 1:** Replicates of nanoparticle tracking analyses (NTA) throughout mouse oviductal organoid cell culture. ‘Passage of Organoids’ refers to the number of times the organoids were removed from their culture dish, broken down into small fragments, and replated to continue growth. The ‘Original Conditioned Medium Volume’ corresponds to the volume of culture medium collected from the organoid cell cultures, and the ‘Volume of Isolated EVs’ refers to the volume of EVs in phosphate buffered saline (PBS) following differential ultracentrifugation for EV isolation from the original conditioned medium.

Replicate	Passage of Organoids	Original Conditioned Medium Volume (mL)	Volume of Isolated EVs (mL)	Concentration (Particles/mL)	Total EVs (# of Particles)	Median EV Size (nm)
**1**	0-4	20	0.82	9.4 x 10^9^	7.7 x 10^9^	134
**2**	5-8	42	0.98	13.2 x 10^9^	12.9 x 10^9^	122
**3**	9-16	72	0.925	11.6 x 10^9^	10.7 x 10^9^	130
**4**	9-20	60	0.93	24 x 10^9^	22.3 x 10^9^	108
**5**	20-23	32	1.46	6.3 x 10^9^	9.2 x 10^9^	139

This protocol uses live mice that recovered from surgery and were used for subsequent experimentation after the ovariectomy. Thus, this protocol may be useful to collect samples that would otherwise be discarded during experimentation, which will result in the reduction of the number of animals used. However, if ovariectomized mice are not needed for other purposes, then tissue collection should be performed as a terminal surgery. Oviductal tissue also may be collected postmortem, although postmortem tissue collection was not tested with this protocol.

**Fig 7:**
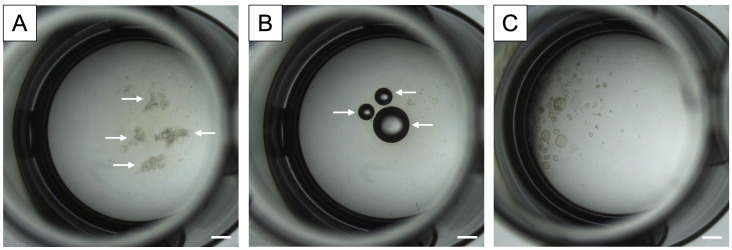
Brightfield micrographs demonstrating errors that may occur while plating mouse oviductal organoids. **A)** Arrows indicate organoid clumping from poor mixing of cell pellet with UltiMatrix prior to plating. **B)** Arrows indicate air bubbles within UltiMatrix droplet. **C)** UltiMatrix droplet containing organoids plated on the edge of the well rather than the center of the well. Scale bar = 1 mm.

Fig 7 shows key considerations for this lab protocol to avoid errors in the organoid culture. If cells are not mixed well in the extracellular matrix (ECM) UltiMatrix, then the resulting organoid development will be in clumps (Fig 7A). If the ECM and cells are mixed too vigorously or quickly, then air bubbles can be introduced (Fig 7B). While air bubbles do not appear to affect the organoid growth, their presence does affect the quality of images captured, i.e., visualizing the organoids in brightfield micrographs is impaired. Lastly, if the droplet of ECM in the culture plate well is not centered, it can spread along the edge of the well (Fig 7C). Again, this does not appear to affect organoid proliferation, but this does affect imaging capability and may disturb the organoids during culture medium changes if the pipette tip is inadvertently placed into the off-center droplet due to its abnormal location.

The provided protocol can be used for experiments such as understanding the normal and pathological physiology of the oviduct and its secreted EVs. The EVs also can be used to understand sperm and embryo interactions with the oviduct and to improve ART outcomes. In conclusion, implementation of this validated lab protocol will result in the long-term viability and proliferation of mouse oviductal organoids and production of their EVs, making it well-suited for laboratories requiring a robust, reliable protocol for reproduction-related research and other fields including cancer biology.

## Supporting information

S1 File Step-by-step protocol, also available on protocols.io. (PDF)

S1 Fig Raw, uncropped Jess simple western blots. (PDF)
