# Supplementary material for: Establishment and culture of mouse oviductal organoids and isolation and characterization of their secreted extracellular vesicles
Source: PLoS One. 2025 Dec 4;20(12):e0337587. doi: 10.1371/journal.pone.0337587 (PMC12677541; doi:10.1371/journal.pone.0337587)
Supplement: S1 File — (PDF) [file pone.0337587.s001.pdf]

Oct 02, 2025

Establishment and culture of mouse oviductal organoids and isolation and characterization of their secreted extracellular vesicles_Supporting Information File S1

DOI
dx.doi.org/10.17504/protocols.io.8epv5kzz4v1b/v1

Riley Thompson[1], Mindy A Meyers[1], Richard McCosh[1], Fiona K Hollinshead[1]
[1]Colorado State University

Riley Thompson
Colorado State University

OPEN ACCESS

DOI: **https://dx.doi.org/10.17504/protocols.io.8epv5kzz4v1b/v1**

Protocol Citation: Riley Thompson, Mindy A Meyers, Richard McCosh, Fiona K Hollinshead 2025. Establishment and culture of mouse oviductal organoids and isolation and characterization of their secreted extracellular vesicles_Supporting Information File S1. **protocols.io https://dx.doi.org/10.17504/protocols.io.8epv5kzz4v1b/v1**

License: This is an open access protocol distributed under the terms of the **Creative Commons Attribution License**, which permits unrestricted use, distribution, and reproduction in any medium, provided the original author and source are credited

Protocol status: Working
We use this protocol and it's working

Created: July 16, 2025

Last Modified: October 02, 2025

Protocol Integer ID: 223005

Keywords: Isoflurane gas, Ovariectomy, Organoid culture, isolation of extracellular vesicle, extracellular vesicle, characterization of extracellular vesicle, oviductal organoid, isolation of oviductal cell, oviductal cell, establishment of organoid, vesicles-supporting information file s1, collection of mouse oviduct, organoids for downstream application, collecting organoid, organoid, mouse oviduct, culture of mouse, cell

Abstract

This protocol details the description of supporting information file S1 which includes preparation and collection of mouse oviducts, isolation of oviductal cells and establishment of organoids, passaging of organoids, collecting organoids for downstream applications, isolation of extracellular vesicles from spent culture medium, and characterization of extracellular vesicles from spent culture medium.

Materials

Equipment:

- Anesthesia machine (VetEquip #901807)

Equipment	
Anesthesia machine	NAME
VetEquip	BRAND
901807	SKU
https://www.vetequip.com/item.asp?cat=&catalogID=901807	LINK

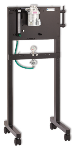

- Anesthesia induction chamber (VetEquip #941444)

Equipment	
Anesthesia induction chamber	NAME
VetEquip	BRAND
941444	SKU
https://www.vetequip.com/item.asp?cat=&catalogID=941443-54	LINK

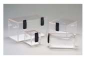

- Intraoperative mouse heating pad (Stoelting #53850M)

Equipment

**Intraoperative mouse heating pad**	NAME
Stoelting	BRAND
53850M	SKU
https://stoeltingco.com/Neuroscience/search?term=53850M&_submit=&_token=aa3df6812.aKEO9xRfAz59sGhxsikcE3ZLeHC6K_lTbi5Wq1bGcRQ.APVIgVYbV3Mb2iM33R1RVwx7EjniSLYAJWcu5C-xPHgJjFaZUmdNRw7zJQ	LINK

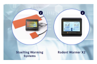

- Postoperative cage warmer (Fisher #NC1867292)

Equipment

**Postoperative cage warmer (Fisher #NC1867292)**	NAME
Fisherbrand	BRAND
NC1867292	SKU
https://www.fishersci.com/shop/products/slide-warmer-29/NC1867292	LINK

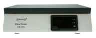

- Clippers (Kent Scientific #CL7300-KIT)

Equipment

Clippers (Kent Scientific #CL7300-KIT)	NAME
Kent Scientific	BRAND
CL7300-KIT	SKU
https://www.kentscientific.com/products/bravmini-professional-cordless-trimmer-kit/?srsltid=AfmBOop0LaCfuBnm13cT8FiBeXVyXqGd5uqVS-V1w1wnb2GMEXcWr2Ii	LINK

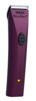

- Surgical scissors (VWR #63042-002)

Equipment

Surgical scissors	NAME
VWR	BRAND
63042-002	SKU
https://www.avantorsciences.com/ca/en/product/9870269/scissors-miniature-self-opening-precision-excelta-corp?isCatNumSearch=true&searchedCatalogNumber=63042-002	LINK

- Thumb forceps (VWR #10198-012)

Equipment

Thumb forceps NAME

VWR BRAND

10198-012 SKU

https://www.avantorsciences.com/ca/en/product/17288495/foerster-iris-tissue-forceps-or-grade-sklar?isCatNumSearch=true&searchedCatalogNumber=10198-012 LINK

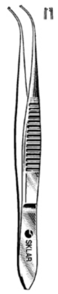

- Dissection microscope (Euromex #NZ.1903-B)
- Micro-scissors (Ambler Surgical #79-582)
- Biosafety cabinet (Labconco #302411101)

Equipment

Biosafety cabinet NAME

Labconco BRAND

302411101 SKU

https://www.labconco.com/product/4-purifier-logic-class-ii-a2-biological-safety-cabinet-with-10-sash-o-54 LINK

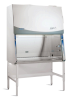

- 20, 200, and 1000 µL Pipettors (ThermoScientific #4641180N, 4641210N, 4641230N)

Equipment

20, 200, and 1000 µL Pipettors NAME

ThermoScientific BRAND

4641180N SKU

https://www.thermofisher.com/order/catalog/product/4641180N LINK

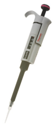

- Microcentrifuge (ThermoScientific #75002447)

Equipment

Microcentrifuge NAME

ThermoScientific BRAND

75002447 SKU

https://www.thermofisher.com/order/catalog/product/75002447?SID=srch-hj-75002447 LINK

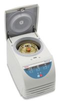

- Inverted microscope (Nikon TMS)
- CO2 incubator (ThermoScientific #51030285)
- Ultracentrifuge (Beckman Coulter Optima XE-90)
- Nanoparticle tracking analyzer (NTA; Particle Metrix ZetaView QUATT)
- Jess ProteinSimple western blot instrument (BioTechne #004-650)

Equipment

Jess ProteinSimple western blot instrument NAME

BioTec BRAND

004-650 SKU

https://www.bio-techne.com/p/simple-western/jess-automated-western-blot-system_004-650 LINK

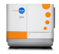

Materials:

- Fluriso Isoflurane liquid (MWI #502017)
- Medical grade oxygen (Airgas OX USPEAMRI)
- VaporGuard Activated Charcoal filter (VWR #89012-608)
- Buprenorphine Base Lab in Polymer Injection Solution (Wedgewood Connect 0.5 mg/mL)
- 4-0 monofilament suture with reverse cutting needle (Vet One #V1-601074)
- Wound clips (Fine Science Tools #12040-01)

Equipment

Wound clips NAME

Fine Science Tools BRAND

12040-01 SKU

https://www.finescience.com/en-US/Products/Wound-Closure/Staple-Systems-Clips/Michel-Suture-Clips/12040-01 LINK

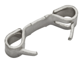

- Betadine (MWI #001574)
- Sterile surgical gloves (MWI #064374)
- No. 10 scalpel blades (VWR #76457-442)
- 35 × 10 mm tissue culture dishes (Corning #353001)

Equipment	
35 × 10 mm tissue culture dishes	NAME
Corning	BRAND
353001	SKU
https://ecatalog.corning.com/life-sciences/b2b/NO/en/Cell-Culture/Cell-Culture-Vessels/Dishes,-Culture/Falcon%C2%AE-Cell-Culture-Dishes/p/353001	LINK

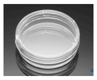

- 48-well culture plates (Corning #3548)

Equipment	
48-well culture plates	NAME
Corning	BRAND
3548	SKU
https://ecatalog.corning.com/life-sciences/b2c/US/en/Microplates/Assay-Microplates/96-Well-Microplates/Costar%C2%AE-Multiple-Well-Cell-Culture-Plates/p/3548	LINK

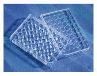

- 20, 200, and 1000 µL pipette tips (ThermoScientific #94420218, 94420318, and 94420813)

Equipment

ClipTip™ Filtered Pipette Tips NAME

Thermo Scientific BRAND

94420813 SKU

https://www.thermofisher.com/order/catalog/product/94420313 LINK

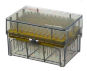

- Microcentrifuge tubes (VWR #490004-444)

Equipment

Microcentrifuge tubes NAME

VWR BRAND

490004-444 SKU

https://www.avantorsciences.com/us/en/product/12610627/genemate-graduated-microcentrifuge-tubes-for-boiling-applications-17-ml-scientific-specialties?isCatNumSearch=true&searchedCatalogNumber=490004-444 LINK

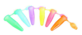

- Ultracentrifuge tubes (Seton Scientific #7022)
- 20 mL syringes (MWI Animal Health #670063)
- 0.22 µm syringe filters (Cell Treat #229747)

Equipment

0.22 µm syringe filters NAME

Cell Treat BRAND

229747 SKU

https://www.celltreat.com/product/229747/?srsltid=AfmBOorUfKDoyHWPwLVFk6IfCsBYQ4HgXx3AzLF0KINR6cI9CHmOcSF9 LINK

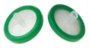

- Cultrex UltiMatrix phenol red-free, growth factor reduced (Biotechne #BME001-05)

Equipment

Cultrex UltiMatrix phenol red-free, growth factor reduced (Biotechne #BME001-05) NAME

Biotechne BRAND

BME001-05 SKU

https://www.bio-techne.com/p/cell-culture/cultrex-ultimatrix-reduced-growth-factor-basement-membrane-extract_bme001-05 LINK

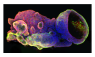

- Phosphate buffered saline (PBS; Accuris Life Science Reagents #EB1200)

Equipment

**Phosphate buffered saline (PBS; Accuris Life Science Reagents #EB1200)**	NAME
Accuris Life Sciences	BRAND
EB1200	SKU
https://accuris-usa.com/?s=EB1200	LINK

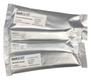

- EV-free PBS (EMD Millipore #TMS-012-A)

Equipment

**EV-free PBS**	NAME
Sigma-Aldrich	BRAND
TMS-012-A	SKU
https://www.merckmillipore.com/IN/en/product/Endotoxin-Free-Dulbeccos-PBS-1X-w-o-Ca-Mg,MM_NF-TMS-012-A	LINK

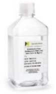

- Organoid Harvesting Solution (Biotechne #3700-100-01)

Organoid Harvesting Solution **Bio-Techne Catalog** #3700-100-01

- 4% paraformaldehyde (ThermoFisher #J19943-K2)
- 2% agarose prepared with water (Bio-Rad #1613101)

2% agarose prepared with water **Bio-Rad Laboratories Catalog** #1613101

- 70% ethanol

- 25% glutaraldehyde (Electron Microscopy Sciences #16220)

25% glutaraldehyde **Electron Microscopy Sciences Catalog** #16220

- Sodium cacodylate trihydrate (Electron Microscopy Sciences #12310)

Sodium cacodylate trihydrate **Electron Microscopy Sciences Catalog** #12310

- Sucrose
- Deionized water
- DMEM/F12 (Gibco #21041-025)

DMEM/F12 (Gibco #21041-025) **Gibco - Thermo Fisher Scientific Catalog** #21041-025

- Parafilm (Millipore Sigma #P6543)

Parafilm (Millipore Sigma #P6543) **Merck MilliporeSigma (Sigma-Aldrich) Catalog** #P6543

- Formvar/carbon supported copper grids (Millipore Sigma #930261)

Equipment

Formvar/carbon supported copper grids	NAME
Millipore Sigma	BRAND
930261	SKU
https://www.sigmaaldrich.com/IN/en/product/aldrich/930261?utm_source=google&utm_medium=cpc&utm_campaign=15000381723&utm_content=129438260635&gad_source=1&gad_campaignid=15000381723&gbraid=0AAAAD8kLQQ79Zkfy0mBTZwuNjjCoOXI2&gclid=EAIalQobChMI6r7y-5nBjgMVly	LINK

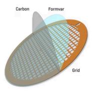

- 2% uranyl acetate (Electron Microscopy Sciences #224002)
- CD9 primary antibody (abcam #ab307085)

CD9 primary antibody **Abcam Catalog** #ab307085

- Hsp70 primary antibody (System Biosciences #EXOAB-Hsp70A-1)
- CYCS primary antibody (Sino Biological #102139-T42)

CYCS primary antibody **Sino Biological Catalog** #102139-T42

- Goat anti-rabbit secondary antibody (Biotechne #042-206)

Goat anti-rabbit secondary antibody **Bio-Techne Catalog** #042-206

- Reagents for Jess western blot (see manufacturer guidelines)

- RIPA Buffer (Sigma-Aldrich #R0278)

RIPA Buffer **Merck MilliporeSigma (Sigma-Aldrich) Catalog #**R0278

- Protease Inhibitor Cocktail Tablets (Roche #11836170001)

Protease Inhibitor Cocktail Tablets **Roche Catalog #**11836170001

- Antibody Diluent 2 (Protein Simple #042-203)

Antibody Diluent 2 **Bio-Techne Catalog #**042-203

- Luminol-S (Protein Simple #043-311)
- Peroxide (Protein Simple #043-379)
- Streptavidin HRP (Protein Simple #042-414)
- EZ Standard Pack (Protein Simple #PS-ST01EZ)
- Protein Normalization Reagent (Protein Simple #043-824)
- Protein Normalization Reconstitution Agent (Protein Simple #043-823)
- 12-230 kDA Fluorescence Separation 8×25 Capillary Cartridges (Protein Simple #SM-FL004-1)

Handling medium:

	A	B	C	D
	**Component**	**Manufacturer**	**Catalog No.**	**Concentration**
	MEM Eagle with Earle's Salts	Sigma	M2279	N/A
	HEPES	Sigma	H0887	25 mM
	Penicillin/Streptomycin	Sigma	P4333	1%
	Sodium pyruvate	Gibco	11360-070	0.1 mM
	Glutamax	Gibco	35050-061	2 mM
	Fetal bovine serum (FBS)	Peak Serum	PS-FB1	20% v/v

MEM Eagle with Earle's Salts **Merck MilliporeSigma (Sigma-Aldrich) Catalog #**M2279

HEPES solution **Merck MilliporeSigma (Sigma-Aldrich) Catalog #**H0887

Penicillin-Streptomycin **Merck MilliporeSigma (Sigma-Aldrich) Catalog #**P4333

Sodium Pyruvate **Gibco - Thermo Fisher Scientific Catalog #**11360070

Glutamax **Gibco - Thermo Fisher Scientific Catalog #**35050-061

Fetal Bovine Serum (FBS) - US Origin **Peak Serum, Inc Catalog #**PS-FB1

Organoid medium:

	A	B	C	D
	**Component**	**Manufacturer**	**Catalog No.**	**Concentration**
	DMEM/F12 without phenol red with L-glutamine	Gibco	21041-025	N/A
	Penicillin/Streptomycin	Sigma	P4333	1%
	B27 Plus	Gibco	A35828-01	2%
	N2	Gibco	17502-048	1%
	Insulin-transferrin-selenium	Gibco	41400-045	1%
	Nicotinamide	Sigma	N0636	1 mM
	Recombinant human EGF	R&D Systems	236-EG	50 ng/mL
	Recombinant human FGF-10	PeproTech	100-26	50 ng/mL
	Recombinant human noggin	R&D systems	6057-NG/CF	100 ng/mL
	TGFβ/Alk inhibitor A83-01	Tocris	2939	0.5 µM
	N-acetyl-L-cysteine	EMD Millipore	106425	1.25 mM
	SB202190	Sigma	S7067	10 µM
	Y27632	EMD Millipore	688000	10 µM

DMEM/F12 (Gibco #21041-025) **Gibco - Thermo Fisher Scientific Catalog #**21041-025

Penicillin-Streptomycin **Merck MilliporeSigma (Sigma-Aldrich) Catalog #**P4333

B-27™ Plus Supplement (50X) **Gibco - Thermo Fisher Scientific Catalog #**A3582801

N2 supplement **Gibco - Thermo Fisher Scientific Catalog #**17502048

Insulin-Transferrin-Selenium (ITS -G) (100X) **Thermo Fisher Catalog #**41400045

Nicotinamide **Merck MilliporeSigma (Sigma-Aldrich) Catalog #**N0636

Recombinant Human EGF Protein CF **R&D Systems Catalog #**236-EG

Recombinant human noggin **R&D Systems Catalog #**6057-NG/CF

TGFβ/Alk inhibitor A83-01 **Tocris Catalog #**2939

N-acetyl-L-cysteine **Merck Millipore (EMD Millipore) Catalog #**106425

SB202190 **Merck MilliporeSigma (Sigma-Aldrich) Catalog #**S7067

Y27632 **Merck Millipore (EMD Millipore) Catalog #**688000

Troubleshooting

Preparation

1 Seek approval for use of research animals from local institution prior to start.

2 Ensure that mouse surgery is conducted in a clean space with adequate ventilation and that charcoal waste gas scavengers are used.

Collect mouse oviducts

3 Induce mouse for surgery using isoflurane gas (up to 5% isoflurane and medical grade oxygen at 3 liters per minute) in an induction chamber.

4 Once mouse is non-reactive, clip fur on dorsal body between shoulders and hips.

5 Position the mouse on a clean surface on top of an intraoperative rodent warmer with the nose of the mouse in a nose cone for inhalant anesthetic (1-5% isoflurane in medical grade oxygen) with periodic monitoring for depth of anesthesia.

6 Apply eye lubricant.

7 Administer buprenorphine (0.012 µL - 0.016 µL of body weight) as an analgesic.

8 Scrub skin with povidone-iodine followed by 70% ethanol three times in the clipped surface.

9 About halfway between the bottom of the rib cage and the hip on the lateral surface of the dorsum, lift the skin with thumb forceps and cut a small incision with surgical scissors.

10 Lift the body wall with thumb forceps, then use surgical scissors to make a small incision into the abdominal cavity.

11 Beneath these incisions, exteriorize the fat pad containing the ovary using thumb forceps.

12 Clamp with hemostatic forceps between the ovary/oviduct and the uterus.

13 Use scissors or scalpel blade to excise the ovary/oviduct. Transfer ovary/oviduct to petri dish containing 1 mL Handling Medium.

14 Release hemostatic forceps while continuing to hold uterine stump with thumb forceps to ensure no bleeding from the uterine stump before releasing tissue back into abdominal cavity.

15 Use suture in a simple interrupted pattern to close the body wall.

16 Close skin with 1 or 2 wound clips.

17 Repeat procedure for opposite ovary.

18 Following bilateral ovariectomy, remove animal from isoflurane and recover in clean cage on a cage warmer. Monitor animals for pain and/or infection over next 7 to 10 days. Remove wound clips 7-10 days post-surgery.

Isolate mouse oviducts using dissection microscope

19 Remove adipose tissue that surrounds mouse ovary and oviduct using thumb forceps and micro-scissors.

20 Dissect the ovary away from the oviduct using micro-scissors or a scalpel blade.

21 Using gentle traction, extend the oviduct from a coiled to straight orientation.

Isolate oviductal cells and establish organoids

55m

22 TIP: Before starting, place UltiMatrix in 4 °C overnight to thaw and place a 48-well plate in 37 °C to warm.

Transfer the isolated oviduct to 1 mL Handling Medium in a new small tissue culture dish.

23 Continuing to use the dissection microscope, use micro-scissors and/or scalpel blade to expose the oviductal lumen.

24 Gently scrape the oviductal lumen to release the cells into the handling medium.

TIP: If too aggressive, oviduct will tear into small pieces that are more difficult to scrape.

Note

ALTERNATIVE OPTION: Co-incubate 1-2 mm sections of isolated oviduct with collagenase to enzymatically isolate the cells.

25 Remove large tissue pieces from the Handling Medium containing the scraped cells.

26 Transfer the cell solution to a microcentrifuge tube.

27 Centrifuge at 600 x g for 00:10:00 at Room temperature . 10m

28 Remove the supernatant.

TIP: Cell pellet may be too small to see but is likely adhered to the bottom/side of the tube. Slowly remove as much supernatant as possible.

29 Add 20X cold UltiMatrix to estimated cell pellet size.

- TIP: If cell pellet cannot be visualized, add 50 µL UltiMatrix.
- TIP: Leave UltiMatrix in 4 °C as long as possible and place on ice when not in 4 °C . Quickly mix the cells with UltiMatrix while avoiding bubbles (bubbles do not appear to affect organoid growth but make imaging more difficult).

30 Place 25 µL droplets of the cells in UltiMatrix in the center of each well in a pre-warmed 48-well plate.

31 Place plate in 37 °C for 00:30:00 - 00:45:00 to allow UltiMatrix droplets to become firmer. 45m

32 Add 250 µL warm (37 °C) organoid culture medium to the wells containing the droplets.

TIP: Slowly run the culture medium down the side of the well rather than on top of the droplet.

33 Add 1 mL PBS to surrounding wells in the plate to reduce evaporation of the culture medium.

34 Replace plate in 37 °C , 5% CO_2 incubator.

35 Remove and replace half of the organoid culture medium every two to three days.

- TIP: Some evaporation will occur-- aim to leave 125 µL of the culture medium in each well. Then add 125 µL of fresh organoid culture medium down the side of the well.
- TIP: Orient pipette tip toward the edge of the well because the droplet containing cells should not be present in that location. Remove spent medium slowly to prevent disruption of the droplet.

Note

For a more uniform number of cells per well, particularly if assessing organoid growth rates, dissociate the organoids into single cells using warm TrypLE Express Enzyme for 00:20:00 with intermittent pipetting. Then count the cells on a hemocytometer using trypan blue 1:1 with the cell solution aliquot. After calculating the live cell concentration, add UltiMatrix to the cell pellet at a ratio that will result in 5,000 or 10,000 cells per well in 25 µL droplets.

Passaging organoids

1h 55m

36 Timing of passage should occur when the organoids are the appropriate size and coloration, which is approximately every 7 days.

TIP: Organoids should be large enough to appreciate a lumen but should be a bright, light color. For mouse oviductal organoids, a dark color is indicative of cellular degeneration.

37 To passage, transfer the contents of each well to a microcentrifuge tube by scraping and removing with a P1000 pipette.

38 Centrifuge at 600 x g for 00:10:00 at Room temperature . 10m

39 Remove supernatant.

TIP: UltiMatrix will be pelleted with the cells. Try not to remove at this time since cells will be removed with the UltiMatrix.

40 Add 200 µL DMEM/F12.

41 Using a P200 pipette set at 150 µL , pipette 500X. Avoid producing bubbles.

42 Add 800 µL DMEM/F12.

43 Centrifuge at 600 x g for 00:10:00 at Room temperature . 10m

44 Remove supernatant. 10m

- TIP: At this point, cells will be in a distinct separate pellet layered below the UltiMatrix. Attempt to remove as much UltiMatrix as possible without disturbing the cell pellet.
- TIP: Can increase centrifugation speed and time to 1000 x g for 00:10:00 if cell pellet is not forming well.

45 Add 200 µL DMEM/F12.

46 Using a P200 pipette set at 150 µL , pipette 300X. Avoid producing bubbles.

47 Add 800 µL DMEM/F12.

48 Centrifuge at 600 x g for 00:10:00 at Room temperature . 10m

49 Remove supernatant.

50 Add 20X cold UltiMatrix to pellet and mix using pipette.

- TIP: Estimate the pellet size and add 20 times more UltiMatrix to the cell pellet. Avoid bubbles.
- TIP: Hold microcentrifuge tube toward the top so that hand is not warming the UltiMatrix.

51 Place 25 µL droplets of the cells in UltiMatrix in the center of each well in a pre-warmed 48-well plate.

52 Place plate in 37 °C for 00:30:00 - 00:45:00 to allow UltiMatrix droplets to become less fluid. 1h 15m

53 Add 250 µL warm (37 °C) organoid culture medium to the wells containing droplets.

TIP: Slowly run the culture medium down the side of the well rather than on top of the droplet.

54 Add 1 mL PBS to surrounding wells in the plate to reduce evaporation of the culture medium.

55 Replace plate in 37 °C , 5% CO_2 incubator.

56 Remove and replace half of the organoid medium every two to three days.

Collecting organoids for downstream applications

7h 10m

57 Flash freeze for RT-qPCR.

57.1 Transfer the contents of each well to a microcentrifuge tube by scraping and removing with a P1000 pipette.

57.2 Centrifuge at 600 x g for 00:10:00 at Room temperature. 10m

57.3 Remove supernatant.

57.4 Add 250 µL cold Organoid Harvesting Solution per each well of organoids that was collected, and place On ice for 01:00:00. 1h

57.5 Centrifuge at 600 x g for 00:10:00 at 4 °C. 10m

57.6 Remove supernatant.

57.7 Add 1 mL DMEM/F12 and resuspend cell pellet.

57.8 Centrifuge at 600 x g for 00:10:00 at 4 °C. 10m

57.9 Remove supernatant.

57.10 Plunge microcentrifuge tube containing cell pellet into liquid nitrogen and transfer to sample box for storage in -80 °C until use for RT-qPCR.

58 Fixation for histology.

58.1 Transfer the contents of each well to a microcentrifuge tube by scraping and removing with a P1000 pipette

58.2 Centrifuge at 600 x g for 00:10:00 at Room temperature . 10m

58.3 Remove supernatant.

58.4 Add 250 µL cold Organoid Harvesting Solution per each well of organoids that was collected, and place On ice for 01:00:00 . 1h

58.5 Centrifuge at 600 x g for 00:10:00 at 4 °C . 10m

58.6 Remove supernatant.

58.7 Add 1 mL PBS and resuspend cell pellet.

58.8 Centrifuge at 600 x g for 00:10:00 at 4 °C . 10m

58.9 Remove supernatant.

58.10 Add 500 µL 4% paraformaldehyde to tube without disturbing cell pellet. Incubate for 00:30:00 at Room temperature . 30m

TIP: Run the paraformaldehyde slowly down the side of the tube.

58.11 Remove the paraformaldehyde without disturbing cell pellet.

58.12 Add 50 µL warm 2% agarose to cell pellet, and transfer the agarose droplet containing the organoids with a P200 pipette with the tip cut off to a petri dish to cool.

TIP: Work quickly or the agarose will cool in the pipette tip.

58.13 After the agarose droplet containing organoids has cooled, transfer to a microcentrifuge tube containing 70% ethanol for storage in 4 °C until use for histology.

59 Fixation for transmission electron microscopy (TEM).

59.1 Transfer the contents of each well to a microcentrifuge tube by scraping and removing with a P1000 pipette.

59.2 Centrifuge at 600 x g for 00:10:00 at Room temperature . 10m

59.3 Remove supernatant.

59.4 Add 250 µL cold Organoid Harvesting Solution per each well of organoids that was collected, and place On ice for 01:00:00 . 1h

59.5 Centrifuge at 600 x g for 00:10:00 at 4 °C . 10m

59.6 Remove supernatant.

59.7 Add 1 mL PBS and resuspend cell pellet.

59.8 Centrifuge at 600 x g for 00:10:00 at 4 °C . 10m

59.9 Remove supernatant.

59.10 Add 500 µL TEM fixative (2% glutaraldehyde with 5% sucrose in [M] 0.1 Molarity (M) sodium cacodylate; pH=7.4) to tube without disturbing cell pellet. Incubate for 02:00:00 at Room temperature . 2h

59.11 Remove the fixative without disturbing cell pellet. 10m

TIP: If the pellet is disturbed, slow centrifuge (200 x g for 00:10:00) can be used to
re-pellet the organoids without damaging them.

59.12 Add 500 µL [M] 0.1 Molarity (M) cacodylate buffer (pH=7.4) for storage in 4 °C until use for TEM.

Isolation of extracellular vesicles from spent culture medium 3h 10m

60 Collect conditioned medium from organoid cell cultures.

61 Centrifuge at 750 x g for 00:10:00 at Room temperature . 10m

62 Remove and save supernatant. Discard pellet.

TIP: Can store supernatant at 4 °C for up to one week before continuing with initial processing.

63 Centrifuge at 3000 x g for 00:10:00 at 4 °C . 10m

64 Remove and save supernatant. Discard pellet.

65 Centrifuge at 17200 x g for 00:30:00 at 4 °C . 30m

66 Remove and save supernatant. Discard pellet.

67 Filter supernatant through a 0.22 µm syringe filter.

TIP: At this stage, can store in -80 °C before continuing EV isolation.

68 Ultracentrifuge samples at 120000 x g for 01:10:00 at 4 °C . 1h 10m

69 Remove and discard supernatant. Leave ~ 0.5 mL pellet.

Note

EV “pellets” often are not visible-be careful when removing supernatants.

70 Add 5 mL EV-free PBS to pellet and mix.

71 Ultracentrifuge samples at 120000 x g for 01:10:00 at 4 °C . 1h 10m

72 Remove and discard supernatant. Leave ~ 0.5 mL pellet, and mix vigorously to ensure EVs are not remaining in pellet stuck to tube.

73 Store the isolated EVs in -80 °C until characterization.

Characterization of extracellular vesicles from spent culture medium 5m

74 Nanoparticle Tracking Analysis of EVs.

74.1 Thaw isolated EVs, if frozen.

74.2 Dilute an aliquot of EVs until ZetaView NTA reads an average particle count of 50 to 500 particles per frame.

74.3 Capture data at 11 positions with instrument in scatter mode.

74.4 Note the particle size distribution and concentration after all data points are captured—be sure to calculate the concentration based on the dilution factor.

Note

1:200 is a good starting EV dilution for NTA.

75 Preparation of EVs for TEM.

75.1 Thaw isolated EVs, if frozen.

75.2 Transfer 30 µL of purified EVs onto parafilm.

75.3 Cover the EV droplets with formvar/carbon supported copper grids. Let incubate at Room temperature for 00:05:00 to allow the grid to absorb the EVs. 5m

75.4 Wash the grid with drops of ddH2O.

75.5 Add 30 µL of 2% uranyl acetate to fix.

75.6 Evaluate the EVs on the grid using a transmission electron microscope.

76 Jess Simple western blot for EVs.

76.1 Prepare each EV sample by combining 3 µL EVs and 1 µL RIPA RTU (1 tablet of protease inhibitor + 1 mL RIPA buffer) per antibody to be evaluated.

76.2 Prepare antibody dilutions. Dilute CD9 antibody 1:25. Dilute Hsp70 antibody 1:50. Dilute CYCS antibody 1:50.

76.3 Use manufacturer protocol to complete Jess Simple Western Blot.
