# Supplementary material for: Establishment and culture of mouse oviductal organoids and isolation and characterization of their secreted extracellular vesicles
Source: PLoS One. 2025 Dec 4;20(12):e0337587. doi: 10.1371/journal.pone.0337587 (PMC12677541; doi:10.1371/journal.pone.0337587)
Supplement: S1 Fig — (PDF) [file pone.0337587.s002.pdf]

Additional File S1 Fig

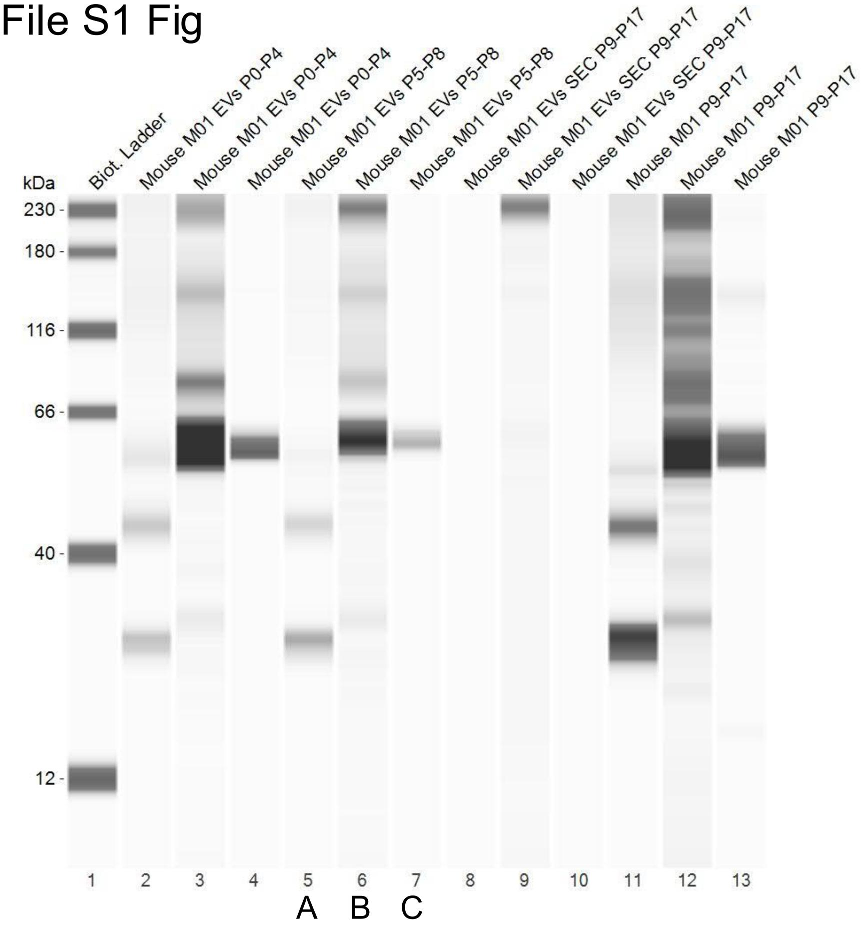

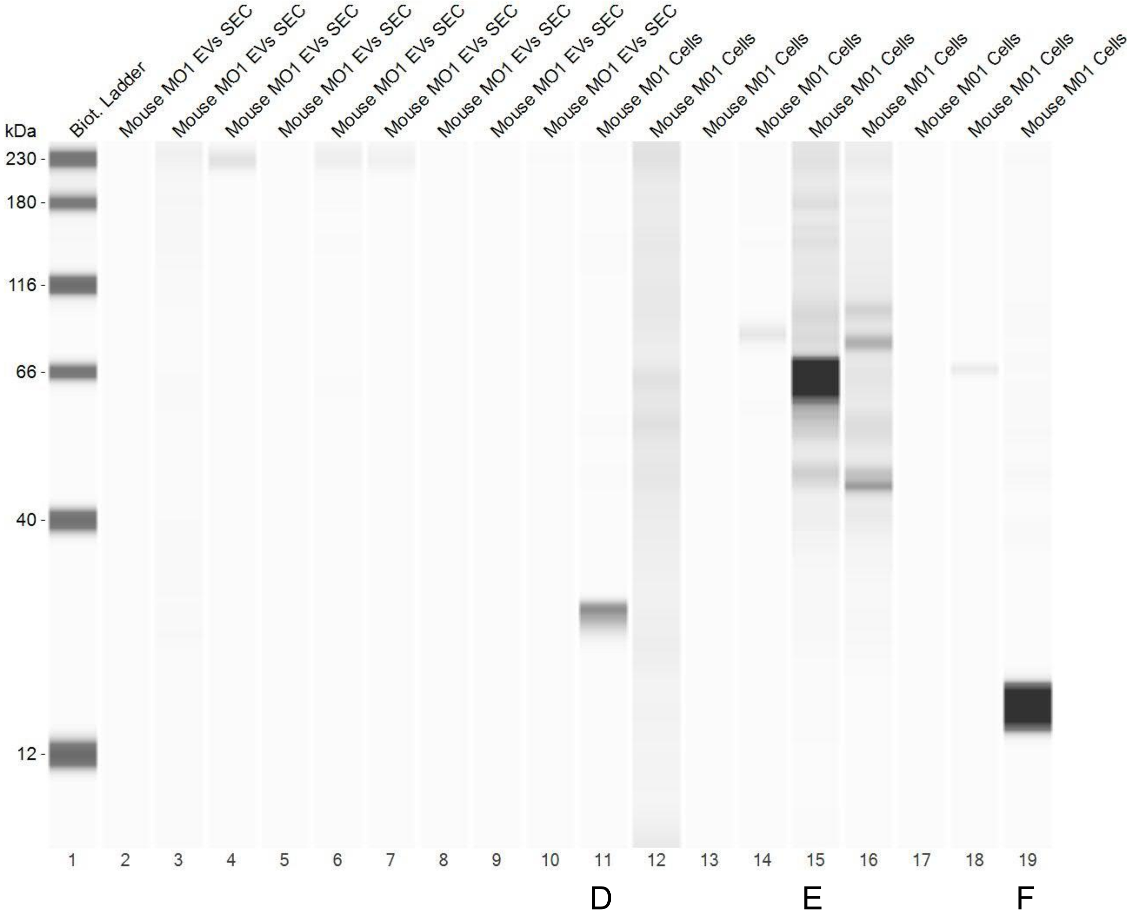

Additional File S1 Fig: Jess simple western blot analysis of extracellular vesicle (EV) and protein markers. A) EVs with CD9 antibody, B) EVs with Hsp70 antibody, C) EVs with CYCS antibody, D) oviductal cells with CD9 antibody, E) cells with Hsp70 antibody, and F) cells with CYCS antibody.
